# Dihydroartemisinin: A Potential Drug for the Treatment of Malignancies and Inflammatory Diseases

**DOI:** 10.3389/fonc.2021.722331

**Published:** 2021-10-07

**Authors:** Ran Yu, Guihua Jin, Manabu Fujimoto

**Affiliations:** ^1^ Department of Immunology and Pathogenic Biology, Yanbian University Medical College, Yanji, China; ^2^ Department of Dermatology, Graduate School of Medicine, Osaka University, Osaka, Japan; ^3^ Laboratory of Cutaneous Immunology, Osaka University Immunology Frontier Research Center, Osaka, Japan

**Keywords:** dihydroartemisinin (DHA), anti-tumor activity, programmed cell death, anti-tumor immunity, inflammatory diseases, pharmacological mechanism

## Abstract

Dihydroartemisinin (DHA) has been globally recognized for its efficacy and safety in the clinical treatment of malaria for decades. Recently, it has been found that DHA inhibits malignant tumor growth and regulates immune system function in addition to anti-malaria. In parasites and tumors, DHA causes severe oxidative stress by inducing excessive reactive oxygen species production. DHA also kills tumor cells by inducing programmed cell death, blocking cell cycle and enhancing anti-tumor immunity. In addition, DHA inhibits inflammation by reducing the inflammatory cells infiltration and suppressing the production of pro-inflammatory cytokines. Further, genomics, proteomics, metabolomics and network pharmacology of DHA therapy provide the basis for elucidating the pharmacological effects of DHA. This review provides a summary of the recent research progress of DHA in anti-tumor, inhibition of inflammatory diseases and the relevant pharmacological mechanisms. With further research of DHA, it is likely that DHA will become an alternative therapy in the clinical treatment of malignant tumors and inflammatory diseases.

## 1 Introduction

Dihydroartemisinin (DHA), a derivative of artemisinin, is the active metabolite of artemisinin-like compounds *in vivo* (1, 2). DHA is widely used in the clinical treatment of malaria and has saved countless lives, due to its 100% efficiency against malaria parasites and low toxicity. DHA kills plasmodium parasites by damaging their membranes, disrupting their mitochondrial function and causing oxidative stress through producing excessive reactive oxide species (ROS) (3, 4). In addition to directly killing malaria parasites, it has recently been found that DHA enhances the ability of the immune system to resist parasites, such as malaria parasites and toxoplasma gondii. Jagannathan et al. find that Ugandan children who receive DHA preventative administration from 6 to 24 months old have a lower incidence of malaria, more IL-2^+^CD4^+^ T cells and TNF-α^+^CD4^+^ T cells, and fewer IL-10^+^CD4^+^ T cells than their peers who do not receive DHA (5). Zhang and colleagues find that DHA increases the number of T-helper (Th) cells and CD8^+^ T cells and the levels of IL-5 and IL-22, decreases the number of B cells and the content of some inflammatory cytokines, including TNF-α, IFN-γ, IL-2, IL-4, IL-6, and IL-10 in the mice who are infected with Toxoplasma gondii or Plasmodium Berghei (6). The above studies show that DHA can not only directly kill parasites, but also prevent hosts from parasitic diseases by regulating T cell subsets and cytokines production.

With the development of DHA research, it has been found that DHA has the pharmacological effect of anti-tumor and inhibition inflammation (7–12). Our laboratory has investigated the anti-tumor and inhibition inflammation effects of DHA and conducts a preliminary analysis of its mechanisms. It has been found that DHA inhibits the growth and metastasis of melanoma in mice by several ways (13). In addition, DHA also significantly alleviates imiquimod-induced mice psoriatic lesions (14). The pharmacological effects of DHA on tumors, pathogen infections, etc. over the past decades have been described in the review of Ho et al. (15), Efferth (16) and Slezakova et al. (17). Therefore, this article will review the last advances in DHA anti-tumor (**Table 1**) and anti-inflammation (**Table 2**) studies in recent years.

**Table 1:** The effects of DHA on tumors.

Tumor	Drug	Effect	Reference
Bladder cancer	DHA	apoptosis↑, ROS↑, p21↑, metastasis↓	(18, 19)
Breast cancer	DHA + Trastuzumab	metastasis↓, AKT signaling pathway↓, ROS↑,	(20)
Breast cancer	DHA + Docetaxel	apoptosis↑, ROS↑, p53↑	(21)
Breast cancer	DHA + Epirubicin	autophagy↑, apoptosis↑	(22)
Breast cancer	DHA + Octreotide + daunorubicin	metastasis↓, VEGF↓, TGF-β↓	(23)
Breast cancer	DHA + Rapamycin	autophagy↑, apoptosis↑, G_0_G_1_ phase arrest	(24)
Breast cancer	DHA	metastasis↓, TGF-β-Smad signaling pathway↓, ROS↑	(25–28)
Cervical cancer	DHA + ALA-PDT	apoptosis↑, ROS↑, NF-κB signaling pathway↓, Nrf2-HO-1 signaling pathway↑	(29)
Cervical cancer	DHA	autophagy↑, apoptosis↑, p-mTOR↓, ROS↑,	(7, 30)
Cholangiocarcinoma	DHA + miR-29b	miR-29b↑, apoptosis↑	(31)
Cholangiocarcinoma	DHA	autophagy↑, apoptosis↑, Akt-mTOR signaling pathway↓	(32)
Colon cancer	DHA + oxaliplatin	ROS↑, G_2_M phase arrest, IFN-γ↑, macrophages↑, dendritic cells↑	(33)
Colon cancer	DHA + Doxorubicin	induction of apoptosis↑, improvement Doxorubicin resistance	(34)
Colon cancer	DHA + 5-fluorouracil	induction of apoptosis↑, improvement 5-fluorouracil resistance	(35)
Colon cancer	DHA	induction of apoptosis↑, MMPs↓, STAT3/p38-MAPK signaling pathway↓	(1, 36, 37)
Endometrial carcinoma	DHA	autophagy↑, apoptosis↑	(38)
Esophageal cancer	DHA	autophagy↑, apoptosis↑, metastasis↓, G_2_M phase arrest, ROS↑, VEGF↓, glycolysis↓,	(8, 39–43)
		Akt-mTOR/NF-κB-HIF-1α/Sonic Hedgehog signaling pathway↓, pyruvate kinase M2↓	
Gallbladder cancer	DHA	TCTP-dependent metastasis↓	(44)
Gastric cancer	DHA + Anlotinib	apoptosis↑, VEGF-A↓, metastasis↓	(45)
Gastric cancer	DHA	apoptosis↑, NF-κB/STAT1/Akt signaling pathway↓, metastasis↓, G_0_G_1_ phase arrest	(36, 46–49)
Glioma	DHA	autophagy↑, apoptosis↑, ferroptosis↑, metastasis↓, ROS↑, miR-21↓, G_0_G_1_ phase arrest, Akt/β-catenin signaling pathway↓	(50–54)
Head and neck squamous cell carcinoma	DHA + Osimertinib	STAT3 signaling pathway↓	(12)
Head and neck squamous cell carcinoma	DHA	apoptosis↑, G_0_G_1_ phase arrest, anti-tumor immunity↑, STAT3 signaling pathway↓	(55, 56)
Hepatocellular carcinoma	DHA + resveratrol	metastasis↓, NF-κB signaling pathway↓	(57)
Hepatocellular carcinoma	DHA + sorafenib	apoptosis↑, ROS↑, G_0_G_1_ phase arrest, TGF-β↓	(58, 59)
Hepatocellular carcinoma	β-dihydroartemisinin-emodin	apoptosis↑, G_0_G_1_ phase arrest	(60)
Hepatocellular carcinoma	DHA	autophagy↑, apoptosis↑, pyroptosis↑, β1,6-branching of N-linked carbohydrate↓, ROS↑,	(61–65)
		glycosylation↓, inhibition MAPK/Akt-mTOR/signaling pathway↓, regulation the	
		gene expression of angiogenesis, apoptosis, cell cycle and signaling pathways	
Human umbilical vein endothelial cell	DHA	autophagy↑, VEFGR1↑, Akt-mTOR signaling pathway↓	(66, 67)
Laryngeal carcinoma	DHA	autophagy↑, metastasis↓, IL-6-STAT3 signaling pathway↓, caspase 1↓, IL-1β↓	(68, 69)
Leukemia	DHA + Navitoclax	apoptosis↑, increase sensitivity to Navitoclax	(70)
Leukemia	DHA	autophagy↑, apoptosis↑, ferroptosis↑, ROS↑, G_0_G_1_ phase arrest,	(71)
Melanoma	DHA	apoptosis↑, NF-κB/STAT3 signaling pathway↓, STAT1 signaling pathway↑, metastasis↓, IFN-γ↑, CTL↑, IL-6↓, IL-10↓, Treg↓	(13, 72]
Multiple Myeloma	DHA	apoptosis↑, ROS↑	(73)
Nasopharyngeal carcinoma	DHA	apoptosis↑, CLC3 chloride channels opening	(11)
Non-small cell lung cancer	DHA + Gefitinib	apoptosis↑, G_2_M phase arrest, Akt-mTOR/STAT3 signaling pathway↓	(74)
Non-small cell lung cancer	DHA +Arsenic Trioxide	apoptosis↑, G_0_G_1_ phase arrest	(75)
Non-small cell lung cancer	DHA + ABT263	apoptosis↑, STAT3/MAPK signaling pathway↓	(76)
Non-small cell lung cancer	DHA	autophagy↑, ferroptosis↑, STAT3/PI3K-Akt/MAPK/β-catenin signaling pathway↓, ROS↑,	(77–79)
		immune escape↓, metastasis↓	
Osteosarcoma	DHA	autophagy↑, ROS↑, p-ERK↑	(80)
Ovarian carcinoma	DHA + curcumin	miR-124↑, G_0_G_1_ phase arrest, apoptosis↑	(56)
Ovarian carcinoma	DHA + Gemcitabine	ROS↑	(9)
Ovarian carcinoma	L-A03	autophagy↑, apoptosis↑, p-JNK↓	(81)
Ovarian carcinoma	DHA	autophagy↑, apoptosis↑, metastasis↓, macrophage↓, G_2_M phase arrest, NF-κB/hedgehog signaling pathway↓	(2, 82–84)
Prostate cancer	DHA	apoptosis↑, G_0_G_1_ phase arrest, glycolysis↓	(85–90)
		IFN-γ↑, Treg↓, γδ T cell↑, perforin↑, granzyme B↑, IFN-γ↑, miR-7↑, miR-34a↑,	
		p-Akt↓, HIF-1α↓,	
NCI-H292, MCF7, HT29, SW480, MEF, HCT116, MDA-MB-453,HT1080	DHA	ferroptosis↑	(91)
A549, HT1080	DHA	ferroptosis↑	(92)

“↑” means increase and “↓” means decrease.

**Table 2:** The effects of DHA in inflammatory diseases.

Model	Drug	Effect	Reference
IgA nephropathy	DHA	mTOR signaling pathway↓, autophagy↑	(93)
Psoriasis	DHA	NF-κB/p38-MAPK signaling pathway↓, CD8^+^ T cells↓, IL-1β↓, IL-6↓, IL-17↓, IFN-γ↓	(14, 94, 95)
Experimental autoimmune encephalomyelitis	DHA	mTOR signaling pathway↓, Th cells↓, Treg↑, TGF-β↑	(96)
LPS-induced neuroinflammation	DHA	PI3K/Akt signaling pathway↓, IL-1β↓, IL-6↓, TNF-α↓	(97)
Inflammatory bowel diseases	DHA	PI3K/Akt/NF-κB-NLRP3 signaling pathway↓, HO-1 signaling pathway↑, Treg↑, Th1, Th17 cells↓,	(98–102)
		IL-1β↓, IL-6↓, IL-23↓, BMD↑, Bacteroidetes↓, Proteobacteria↑ Verrucomicrobia↓, Firmicutes↑,	
Rheumatoid arthritis	DC 32	Nrf-2/HO-1 signaling pathway↑, Th17 cells↓, Treg↑, IL-6↓, MMPs↓, RF↓	(103, 104)
Osteoarthritic synovium	DC 32	Nrf-2/HO-1 signaling pathway↑, IL-1β↓, IL-6↓, MMPs↓, CXCL12↓, CX3CL1↓,	(105)
Systemic lupus erythematosus	DHA	Nrf-2/HO-1 signaling pathway↑, TNF-α-TLR4-NF-κB signaling pathway↓, Th17↓, Treg↑, IFN-α↓,	(106–108)
		IFN-β↓, IL-1β↓, IL-6↓	
Lupus nephritis	DHA + siHMGB1	TNF-α-TLR-4-NF-κB signaling pathway↓, HMGB1↓, IL-1β↓, IL-6↓	(109)
LPS-induced acute kidney injury	DHA	NF-κB signaling pathway↓, IL-1β↓, IL-6↓, IL-17A↓, TNF-α↓, IFN-γ↓, CXCL-1↓	(110)
Allergic Asthma	DHA	IL-6-STAT3/p38-MAPK/NF-κB signaling pathway↓, OVA-specific IgE↓, miR-183C↓, Th17 cells↓,	(111, 112)
		IL-1β↓, IL-4↓, IL-17↓, IL-21↓, GM-CSF↓, IL-10↑	
Human bronchial epithelial cell	DHA	CBF/NFY↑, FOXO1↑, HSF↑, Smad↑, SRF↑, STAT3↑, Nrf-2↑	(113)
LPS−induced acute lung injury	DHA	TNF-α-NF-κB signaling pathway↓, macrophages↓, neutrophils↓, Nrf-2↑, IL-1β↓, IL-6↓	(114)

“↑” means increase and “↓” means decrease.

## 2 The Anti-Tumor Activity of DHA

Malignant tumors threaten the life safety of people all over the world and have become the main cause of death together with cardiovascular and cerebrovascular diseases and respiratory diseases (115). It has been demonstrated that DHA exerts significant anti-tumor activity in a variety of malignant tumors, and it has no toxicity to normal cells at appropriate doses (18, 20, 85, 94, 116, 117). It has been found that DHA not only is used as an adjuvant drug in combination with other chemotherapy drugs to improve drug resistance and enhance tumor killing function, but also has a significant inhibitory effect on tumors (21, 29, 34, 39, 45, 46, 70). Gao et al. find that combination of DHA and resveratrol inhibits the proliferation, metastasis and invasion of Hep2 and MDA-MB-231 cancer cells (57). Chen et al. confirm that DHA alleviates the resistance of multiple myeloma to dexamethasone (73). Hu and colleagues demonstrate that DHA/miR-29b combination therapy significantly inhibits proliferation and promotes apoptosis of cholangiocarcinoma cells (31). Guo et al. find that DHA increases the sensitivity of drug-resistant breast cancer cells to chemodynamic therapy, and plays a role in promoting chemotherapy as an adjuvant therapy drug (25). Similar to the mechanism by which DHA kills plasmodium parasites, DHA causes oxidative stress in tumor cells by lysing the peroxide groups, then leading tumor to death (55). In addition, DHA also plays an anti-tumor role through inhibiting tumor metastasis, inducing programmed cell death, blocking cell cycle, and enhancing anti-tumor immunity.

### 2.1 Inhibition of Tumor Metastasis

Tumor metastasis is one of the main causes of tumor treatment failure. Epithelial-mesenchymal transformation (EMT), matrix metalloproteinases (MMPs) secretion and neovascularization are closely related to tumor metastasis. DHA has exerted anti-metastasis effect in a variety of tumors (26, 44, 47, 50, 68, 118). In our study, DHA inhibits the melanoma metastatic capacity by reducing the production of MMPs *in vivo* and *in vitro* (13). Furthermore, DHA significantly reduces the number of pulmonary melanoma nodules, inhibits EMT, and decreases the expression of MMPs in the mouse melanoma lung metastasis model (Data not shown). Li et al. find that DHA inhibits EMT of ovarian cancer, thereby reducing its metastasis to the lung, liver and intestine (82). TGF-β-Smad signaling pathway and Akt signaling pathway play important roles in DHA suppressing tumor metastasis. In breast cancer, DHA inhibits EMT by reducing TGF-β production and decreasing phosphorylation of Samd2 and Smad3 (27). DHA inhibits phosphorylation of Akt in glioblastoma cells, thereby inhibiting EMT and reducing MMPs secretion (51). Similarly, Ju et al. observe that DHA inhibits breast cancer metastasis by reducing the production of MMPs, vascular endothelial growth factor (VEGF), and TGF-β (23).

As metastatic tumors grow, they induce angiogenesis by upregulating the expression of HIF-1α and VEGF to combat the lack of oxygen and nutrients within the tumor. It has been shown that DHA induces autophagy in human umbilical vein endothelial cells by inhibiting Akt and mTOR signaling pathways, thereby inhibiting their ability to generate blood vessels *in vitro* (66). In addition, DHA also inhibits angiogenesis by increasing the expression of VEFGR1, a deceptive receptor of VEGF, to block the binding of VEGF and VEFGR2 (67). Li et al. find that DHA inhibits esophageal cancer growth and metastasis by inhibiting the phosphorylation of p65 and reducing the activity of HIF-1α and VEGF (40). These suggest that DHA has a strong inhibitory effect on angiogenesis.

### 2.2 Induction of Programmed Cell Death in Tumors

#### 2.2.1 Apoptosis

Apoptosis, a kind of programmed cell death, is the main way that drugs cause tumor cell death and is regulated by many factors, such as Bcl-2 protein family (12, 35, 48). In non-small cell lung cancer (NSCLC), DHA inhibits the expression of anti-apoptotic Bcl-2, disrupts the balance of the Bcl-2 family, and induces apoptosis (76). Similarly, in cervical cancer, DHA regulates Bcl-2 family protein expression and induces HeLa cell apoptosis by inhibiting the phosphorylation of mTOR (30).

In addition to changing the balance of members of the Bcl-2 protein family, DHA causes the accumulation of ROS in tumor cells, leading to mitochondrial damage (61). With excessive damage of mitochondrial, caspase cascade is activated and eventually activates caspase 3, the executor of apoptosis, causing mitochondrial apoptosis (19). Im et al. find that DHA increases the expression of cleaved caspase 8, cleaved caspase 9 and cleaved caspase 3 by inhibiting MAKP signaling pathway in hepatocellular carcinoma, thus inducing caspase-dependent apoptosis (62). Li et al. use β-dihydroartemisinin-Emodin to treat human liver cancer HepG-2 cells and similar results are observed. β-dihydroartemisinin-Emodin activates caspase 8, caspase 9 and caspase 3 and induces apoptosis by regulating the balance of Bcl-2/Bax (60). In our study, DHA increases the ratio of Bax/Bcl-2, activates caspase cascade and induces mitochondrial apoptosis by regulating STAT3 and other signaling pathways, thereby inhibiting mouse melanoma (13). In addition, DHA also induces endoplasmic reticulum stress (119). In glioblastoma cells, DHA not only induces mitochondrial apoptosis mediated by cleaved caspase 9 and cleaved caspase 3, but also induces endoplasmic reticulum stress pathway of apoptosis by activating caspase 12 (52). Interestingly, Luo et al. demonstrate that DHA induces endoplasmic reticulum stress by activating PERK in porcine ovarian cancer (120). These results indicate that DHA has a wide range of anti-tumor effects, not only its pharmacological mechanisms are diverse, but also has obvious anti-tumor effects in many species.

#### 2.2.2 Autophagy

In addition to inducing apoptosis, DHA also has the ability to induce autophagy in tumors (22, 41, 69, 77, 81). When DHA damages tumor cells, autophagy is activated to clear damaged organelles, but excessive autophagy often leads to cell death. During autophagy, a series of ATG proteins is activated, and microtubule-associated protein 1 light chain 3-I (LC3-I) is transformed into LC3-II to form the membrane structure of autophagosome (24). In cholangiocarcinoma and hepatocellular carcinoma, DHA induces autophagy by inhibiting the Akt-mTOR signaling pathway (32, 63). Further, Tang et al. find that DHA up-regulates LC3-II/LC3-I ratio and induces autophagy in endometrial cancer and cervical cancer cells (38).

#### 2.2.3 Ferroptosis

Ferroptosis is a type of programmed cell death different from apoptosis and autophagy. In ferroptosis, excessive intracellular free iron reacts with lipid peroxides and produces a large amount of ROS, which causes cell damage and death after depletion of intracellular glutathione (121). In osteosarcoma, Shen et al. demonstrate that DHA induces autophagy and mitochondrial injury by increasing the content of ROS, and the above effects are associated with iron (80). Furthermore, it has been found that DHA induces ferroptosis by increasing ROS level in tumor cells (53). Yuan et al. confirm that DHA induces ferroptosis in NSCLC cells by regulating SLC7A11 expression, accompanied by a decrease in intracellular GSH content and an increase in ROS and MDA (78). Du et al. find that DHA induces ferroptosis in leukemia cells by autophagy mediated degradation of ferritin and subsequent increases of intracellular free iron (71). However, Chen et al. use DHA to treat lung cancer cells, colon cancer cells, breast cancer cells and other tumor cells and observe that DHA can also induce ferroptosis in the above cells, but the mechanism is not related to autophagy (91). Interestingly, Bai and colleagues find that the rearrangement of DHA to monoketo-aldehyde-peroxyhemiacetal under physiological significantly enhance its response to iron and this physiological process is pH-dependent (92). The above studies suggest that DHA induces ferroptosis in many tumor cells mainly through promoting ROS production. However, the underlying mechanisms, such as the role of autophagy in DHA-induced ferroptosis, remain controversial and require further research.

### 2.3 Tumor Cell Cycle Arrest

Cell cycle progression requires interaction between cyclins and cyclin-dependent kinases (CDKs). In tumors, abnormal expression of cyclins and CDKs leads to dysfunction of cell cycle checkpoints and rapid tumor growth (36, 75). It has been shown that DHA blocks tumor cell cycle at G0/G1 or G2/M checkpoints by regulating the expression of cyclins and CDKs (42, 54, 74, 86). Zhao et al. find that combination treatment with DHA and curcumin have the effect of up-regulating the expression of miR-124 and blocking cell cycle at G0/G1 phase in ovarian carcinoma (56). Du et al. treat leukemia cells with DHA and observe that CDK2, CDK4 and Cyclin D expression are significantly inhibited and cell cycle is arrested at G0/G1 phase in the DHA treatment groups (71). Li et al. use ART or DHA treated ovarian carcinoma cells and find that DHA show a stronger anti-tumor effect and both ART and DHA block cell cycle at G2/M phase (83).

### 2.4 Enhance Anti-Tumor Immunity

In tumor microenvironment, CD8^+^ cytotoxic T lymphocytes (CTLs) play a major role in killing tumor cells. However, tumor cells and regulatory T cells (Tregs) suppress the killing effect of CTLs by secreting IL-10 and TGF-β, thus realizing tumor immune escape (122). It has been shown that DHA inhibits the production of TGF-β (23, 26–28) and TNF-α (49) in many tumors. Noori et al. observe that, in pancreatic cancer, DHA has the function of inhibiting Treg and increasing IFN-γ production in tumor microenvironment (87). In pancreatic cancer, Zhou et al. find that DHA enhances the activity of T cells and promotes the secretion of perforin, Granzyme B and IFN-γ (88). In our study, DHA exerts a significant modulation of anti-melanoma immunity. After DHA treatment, the levels of IL-6 and IL-10 in serum and melanoma of melanoma bearing mice are significantly decreased, while IFN-γ is significantly increased. Furthermore, DHA enhances the killing effect of CTLs to mouse melanoma by reducing IL-10 content and the number of Treg in tumor microenvironment (13). In melanoma lung metastasis models, DHA reduces the number of infiltrating Tregs in lung tissue and increases the infiltration and function of CTLs (Data not shown).

In addition to regulating the number and function of T cell subsets, DHA can also regulate other immune cells to promote anti-tumor immunity. Dendritic cells and macrophages are also involved in anti-tumor immunity. However, depending on their phenotypes, they can either inhibit or promote tumors (123, 124). Chen et al. confirm that DHA inhibits the differentiation of THP-1 cells to M2 macrophage and plays an anti-tumor role by inhibiting the phosphorylation of STAT3 (125). It has been demonstrated that DHA inhibits ovarian cancer metastasis by reducing the production of MMPs and the infiltration of macrophages in the metastatic sites of ovarian cancer (84). Furthermore, in colorectal tumor, DHA significantly strengthens anti-tumor immunity by enhancing the phagocytosis function of dendritic cells and macrophages and promoting the production of IFN-γ by T cells. The above effects are more obvious when DHA is combined with anti-PD-L1 mAb (33). Further, Zhang et al. confirm that in NSCLC, on the one hand, DHA reduces the expression of PD-L1 on tumor cells by inhibiting the expression of TGF-β and PI3K/Akt and STAT3 signaling pathways, thus impairs tumor immune escape. On the other hand, DHA increases the sensitivity of NSCLC to radiotherapy by regulating the expression of EMT-associated proteins (79). In summary, DHA promotes T-cell-centered anti-tumor immunity by reducing the secretion of immunosuppressive cytokines and suppressing the number and function of immunosuppressive cells.

### 2.5 Other Anti-Tumor Effects

The detection and analysis of abnormal expression of genes, proteins and metabolites in tumors are helpful to identify tumor pathogenesis and explore effective tumor treatment methods. Hepatocellular carcinoma MHCC97-L cells are treated with DHA for Global gene expression profiles analysis. The results show that DHA regulates gene expression which is associated with angiogenesis, apoptosis, cell cycle and various pathways (64). As a kind of non-coding RNA, Micro-RNA (miR) plays an important role in tumor by connecting to mRNA to regulate gene expression (72). Paccze et al. demonstrate that DHA inhibits tumor growth by up-regulation miR-7 and miR-34a in prostate cancer (90). In addition, Zhao et al. find that DHA binding curcumin up-regulate miR-124 and inhibit ovarian cancer (56). Hou et al. treat hepatocellular carcinoma cells MHCC97H and HCCLM3 with DHA and Sorafenib, and perform a proteomics analysis using Tandem Mass Tag peptide coupled with LC-MS/MS. Compared with the control group, there are 532, 426 and 628 differentially expressed proteins in the DHA group, Sorafenib group and DHA + Sorafenib group, respectively. And these differentially expressed proteins are mainly involved in cellular component organization, response to stress, and intracellular biochemical and metabolic reactions (58). Further, the metabonomic analysis of DHA in the treatment of esophageal cancer is performed. The results show that DHA down-regulates pyruvate kinase M2 and inhibits glycolysis, which is the main way for cancer cells getting energy (43). Zhu et al. demonstrate that DHA also inhibits glycolysis in prostate cancer (89). Liu et al. conduct a network pharmacologic analysis for the anti-hepatocellular carcinoma effect of DHA and find that DHA regulates the glycosylation and inhibits liver cancer by downregulating the expression of β1,6-branching of N-linked carbohydrate (65).

**Figure 1:**
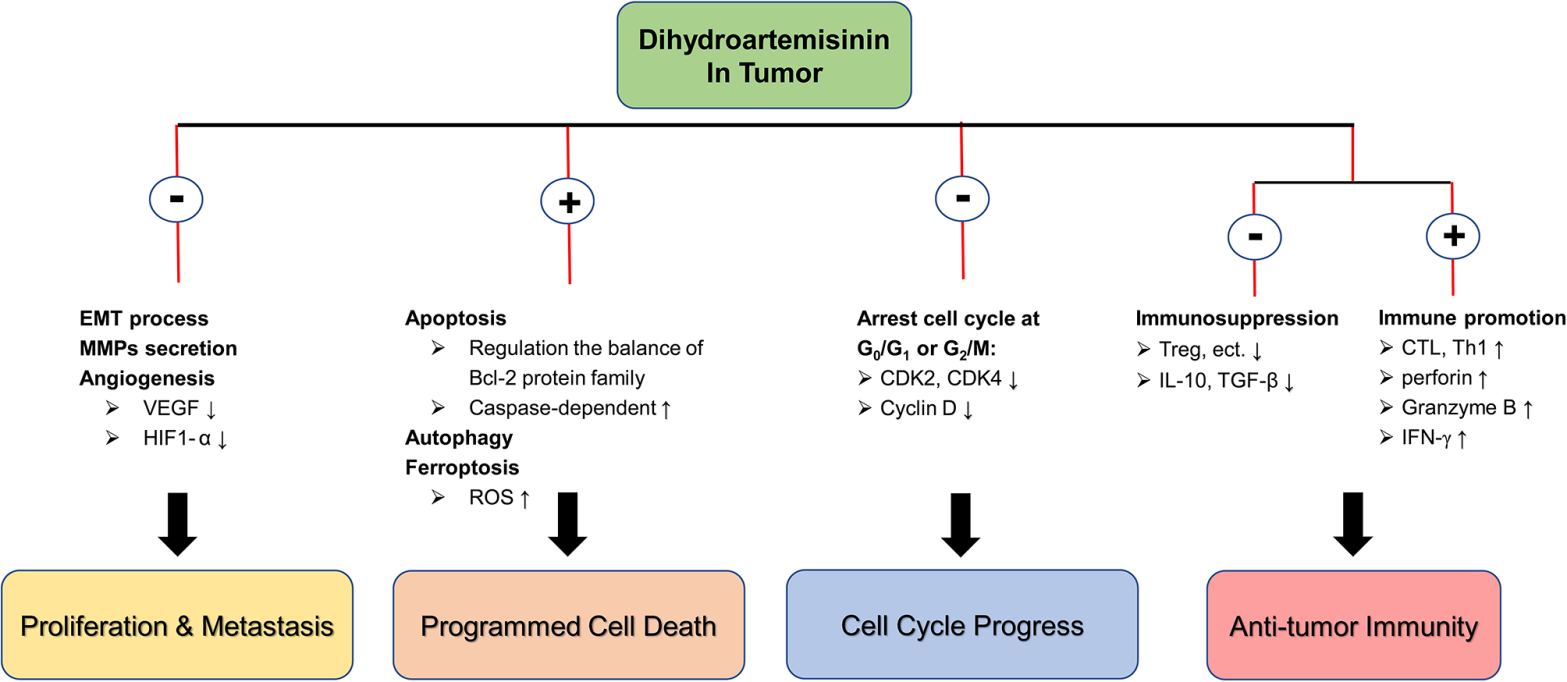
The molecular mechanism of DHA in anti-tumor. ⊕ means the promoting effect of DHA. ⊖ means the suppressive effect of DHA.

In summary, DHA has significant inhibitory effects on a variety of tumors in different species, and its pharmacological mechanisms are diverse, including inhibition of cell proliferation and metastasis, induction of autophagy and multiple programmed cell death, and arrest of cell cycle, etc. In addition, DHA exerts the strong effect in promoting anti-tumor immunity. DHA corrects the immunosuppression state of tumor-bearing mice by reducing the secretion of IL-10 and TGF-β and suppressing the number and function of Treg, and then activate the anti-tumor immune response mediated by T cells with CTL as the main body. In particular, DHA is not toxic to normal cells and is a potential tumor suppressive option (**Figure 1**).

## 3 The Anti-Inflammation Activity of DHA

It has been shown that DHA regulates the immune function in the treatment of parasitic diseases and tumors (5, 6, 13, 33, 84, 87, 88, 125). In addition, it also has been reported that DHA significantly inhibits inflammatory diseases. As described by Xia et al. in IgA nephropathy mesangial cells (IgAN HMCs) induced by aggregated IgA1, DHA promotes autophagy of IgAN HMCs by down-regulating mTOR expression, and thus exerts an anti-proliferation effect (93). Further, as a potential anti-inflammatory drug, DHA plays a powerful role in inhibiting a variety of inflammation-related diseases. The potential pharmacological mechanisms of DHA anti-inflammation are described by following and list in **Table 2**.

### 3.1 Psoriasis

Psoriasis is a chronic inflammatory skin disease characterized by excessive proliferation of keratinocytes and excessive infiltration of white blood cells (e.g., neutrophils, T cells, etc.) into the dermis. As described by Wei et al., we find that DHA significantly alleviates imiquimod-induced psoriasis-like skin inflammation in mice and inhibits keratinocyte proliferation by regulating NF-κB and p38-MAPK signaling pathways (14). Furthermore, the content of IL-1β, IL-6 and IL-18 decrease significantly with the DHA treatment. In particular, the expression of CXCL-1 is markedly reduced, suggesting that DHA has a potential ability to inhibit neutrophil chemotaxis. In addition, our study also shows that DHA has a potent regulatory effect on a variety of immune cells, such as T cells, and pro-inflammatory cytokine production (94). The imbalanced proportion of T cell subsets leads to the skin immune system overactivation, which is considered to be closely related to psoriasis. Chen and colleagues demonstrate that DHA relieves mice psoriatic-like skin lesions and prevents psoriasis recurrence (95). Specifically, DHA treatment diminishes the number of pathogenic CD8^+^ central memory T cells and CD8^+^ resident T cells in the skin and reduces the content of pro-inflammatory cytokines such as IL-17. Further, the expression of eomesodermin and BCL-6 in CD8^+^ T cells is also down-regulated with DHA treatment.

### 3.2 Experimental Autoimmune Encephalomyelitis (EAE)

EAE is an autoimmune disease mediated by specific CD4^+^ T cells, characterized by mononuclear cell infiltration into the central nervous system and demyelination of white matter (126). Zhao et al. treat EAE mice with DHA and find that the symptoms are significantly improved and disease scores decrease markedly. The mechanism is that DHA increases the number of Treg in the spinal cord, spleen, inguinal lymph nodes and peripheral lymph nodes, promotes the differentiation of Treg and the production of TGF-β and reduces Th cell infiltration through inhibiting mTOR signaling pathway (96). In addition, DHA alleviates LPS-induced neuroinflammation by inhibiting PI3K/Akt signaling pathway and reducing the production of IL-1β, IL-6 and TNF-α (97).

### 3.3 Inflammatory Bowel Disease (IBD)

It has been reported that the pathogenesis of IBD is closely related to the over-activation of several signaling pathways (such as NF-κB signaling pathway and p38-MAPK signaling pathway), the production of pro-inflammatory cytokines and abnormal intestinal mucosal immune system (127). Li et al. find that DHA alleviates IBD in mice by inhibiting PI3K/Akt and NF-κB signaling pathways and reducing the production of TNF-α, IL-1β and IL-6 (98). It has been found that NLRP3 inflammasome, which acts as a bridge between some inflammation-related signaling pathways and pro-inflammatory cytokines, is often overexpressed in IBD. Liang et al. find that in IBD mice, DHA inhibits the phosphorylation of p65 and p38, down-regulates the expression of NLRP3 inflammasome, and thereby reduces the contents of IL-1β, IL-6 and TNF-α (99). Further to explore the regulatory effect of DHA on immune cells, Yan and colleagues demonstrate that DHA induces T cell apoptosis *via* HO-1, thus reducing the number of Th1, Th9 and Th17 cells, restoring the balance between Th cells and Treg, and improving IBD symptoms (100). In addition to reducing inflammatory cell infiltration and pro-inflammatory cytokine production in the gut, DHA also improves IBD by regulating intestinal flora. Lei et al. use 16S rDNA gene analysis and find that DHA restores the abundance of Bacteroidetes, Verrucomicrobia, Firmicutes and Proteobacteria, which are abnormal in mouse IBD (101). Interestingly, DHA exerts a significant inhibitory effect on IBD-induced bone loss by reducing osteoclast formation and increasing bone mineral density (102). The inhibitory effect of DHA on osteoclasts is not limited to IBD. In breast cancer (128) and other diseases (129, 130), DHA inhibits the formation of osteoclasts and relieves bone loss, suggesting that DHA seems to have a relieving effect on bone loss caused by a variety of diseases. DHA is expected to become an alternative therapeutic drug for the treatment of osteolytic bone disease.

### 3.4 Rheumatoid Arthritis (RA)

RA is an autoimmune disease characterized by extensive production of autoantibodies and severe damage of joints (131). Fan et al. use DC32, a derivative of DHA, to treat collagen-induced arthritis mouse, and find that after DHA treatment, the content of rheumatoid factor significantly decreases, cartilage damage is alleviated and arthritis is improved. Its mechanism is that on the one hand DC32 activates nuclear factor (erythroid-derived 2)-like 2 (Nrf-2)/HO-1 antioxidant pathway and on the other hand DC32 reduces IL-6 transcription and restores Treg/Th17 cells balance (103, 104). In addition, Li et al. demonstrate that DC32 reduces the expression of IL-6, IL-1β, TNF-α, CXCL12 and CX3CL1 by activating Nrf-2/HO-1 signaling pathway and inhibiting the phosphorylation of ERK and p65, thus improving Osteoarthritic Synovium (105).

### 3.5 Systemic Lupus Erythematosus (SLE)

SLE is a chronic autoimmune disease characterized by the production of autoantibodies and the involvement of multiple organs (132). Following the discovery of DHA in malaria, Tu Youyou’s team finds that DHA has a significant inhibitory effect on SLE. In BXSB mice, DHA significantly reduces the production of TNF-α by inhibiting the expression of p65 in peritoneal macrophages and renal tissue (106). In addition, Huang et al. confirm that DHA inhibits Toll-like receptor 4 (TLR4) and reduces IRF3 and type I interferon (IFN-α and IFN-β) production, thus alleviates SLE (107). Furthermore, Li et al. find that DHA delays the progression of SLE by inhibiting the senescence of myeloid-derived suppressor cells. In addition, DHA restores Treg/Th17 cells balance and reduces serum IL-1β, IL-6 and IL-8 levels in lupus mice (108).

Lupus nephritis is a complication of SLE and often leads patients to death. Diao et al. use LPS stimulation glomerular mesangial cell line MMC to build cell model of lupus nephritis and give DHA treatment. After DHA treatment, the expression of HMGB1, one of the genes closely associated with lupus nephritis, is significantly reduced. In addition, DHA attenuates the expression of TLR4 and p65 and reduces IL-1β, IL-8, IL-6 and TNF-α production in LPS-induced RAW264.7 cells (109). In LPS-induced acute renal injury, DHA therapy reduces the serum Scr and BUN levels and restores renal function. The mechanism is that DHA reduces the expression of IL-1β, IL-5, IL-6, IL-17A, TNF-α, IFN-γ, CXCL-1 and improves oxidative stress through inhibiting the phosphorylation of p65 (110).

### 3.6 Allergic Asthma

Allergic Asthma is a chronic airway allergic disease. It has been reported that Th2 cells, Th17 cells, mast cells and granulocytes are all involved in the pathogenesis of Asthma (133). Wei et al. find that DHA significantly reduces inflammatory cell infiltration and alleviates asthma-related airway hyper-responsiveness in ovalbumin (OVA)-induced mouse asthma. The mechanism is that DHA inhibits the activation of ERK, p38 and NF-κB and reduces the OVA-specific Ig E and Th2 cytokines secretion (111). In addition to the above effects, Zhu et al. demonstrate that DHA also relieves asthma by reducing the number of Th17 cells and the production of IL-17, IL-21, IL-22, IL-1β, TNF-α and GM-CSF (112). Furthermore, Ravindra et al. use Untargeted Proteomics to analyze the targets of DHA treatment human bronchial epithelial cells and find that DHA promotes the expression of forkhead box protein O1 (FOXO1), Nrf-2, serum response factor (SRF), STAT3 and Smad (113). For other lung injury diseases, DHA reduces the release of IL-1β, IL-6 and TNF-α by inhibiting the phosphorylation of p65 and activating Nrf-2 in LPS-induced acute lung injury. In addition, DHA also reduces the infiltration of macrophages and neutrophils into lung tissue to alleviate lung injury (114).

**Figure 2:**
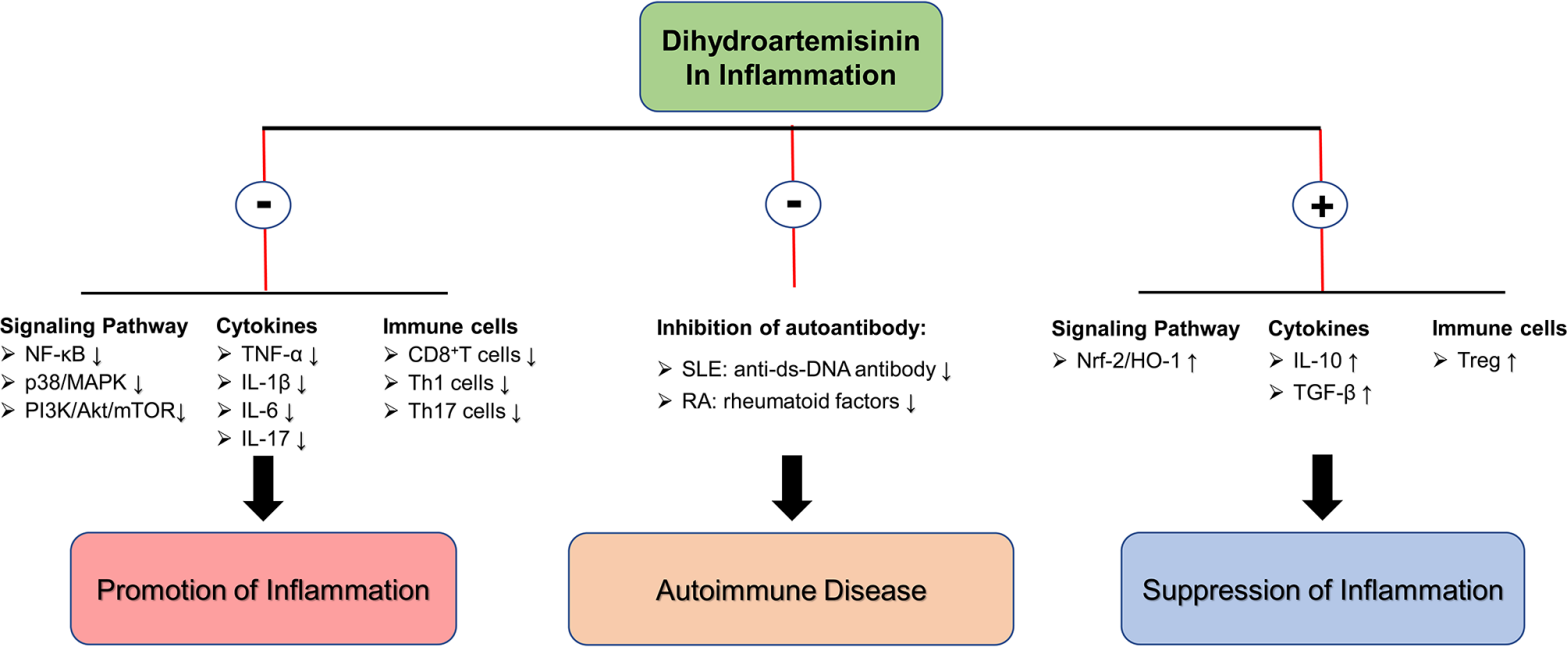
The molecular mechanism of DHA in inflammation inhibition. ⊕ means the promoting effect of DHA. ⊖ means the suppressive effect of DHA.

In inflammatory diseases, an unbalanced immune system forms a complex inflammatory immune network. The insufficiency of Treg cell function leads to the continuous development of inflammation with Th1/Th17 cells as the core. The release of pro-inflammatory cytokines producing by Th1/Th17 cells further inhibits the function of Treg. It forms a closed-loop structure of positive feedback, which leads to the continuous development of inflammatory disease and prolonged healing. DHA and its derivative DC32 have shown alleviative effects in multiple inflammatory diseases by regulating the signaling pathways (inhibiting pro-inflammatory signaling pathways and activating antioxidant signaling pathways), inhibiting pro-inflammatory cytokine production (such as TNF-α, IL-1β, etc.), and restoring immune cell balance. In addition to the aforementioned effect, in autoimmune diseases, DHA may also relieve symptoms and inhibit progression by reducing the production of autoantibodies. In laboratory studies, researchers have tentatively demonstrated the potential of DHA as an alternative treatment for inflammatory diseases, and further exploration of the underlying mechanisms of DHA inhibiting inflammation and the clinical use of DHA in treating inflammation needs to be performed (**Figure 2**).

## 4 Summary and Future Outlook

With the development of anti-tumor and inhibition of inflammation effects of DHA, in recent years, more and more studies have focused on how to improve the efficacy of DHA. The main strategies to enhance the efficacy of DHA include modifying the chemical structure of DHA (55), developing new DHA derivatives (134), building multimeric DHA conjugates (135), and coupling other drugs with DHA to achieve targeted tumor therapy (37, 59, 136–138). Liu et al. construct the R_8_ modified epirubicin-dihydroartemisinin liposomes for the treatment of NSCLC, and find R_8_ modified epirubicin-dihydroartemisinin liposomes have a stronger killing effect on A549 cells. R_8_ modified epirubicin-dihydroartemisinin liposomes attenuate the metastasis of A549 cells by inhibiting the production of TGF-β, MMP2 and HIF-1α. *In vivo*, R_8_ modified epirubicin-dihydroartemisinin liposomes accumulate in tumor and show the targeted functions, indicating that it is an effective treatment method for NSCLC (139).

Moreover, DHA and artemisinin-based drugs are also increasingly reported in clinical oncology trials. For clinical treatment, DHA is widely used in the treatment of malaria which provides a solid and effective basis for its safety. In laboratory studies, appropriate concentration of DHA has low toxicity to normal cells, but some studies have reported that even though DHA is a high safety drug, excessively high concentration of DHA (≥100μmol/L) can still cause damage to normal cells (140, 141), which also suggests that the clinical concentration of DHA should not be too high. And, when using large doses of DHA, clinician should be aware of its toxic effects. Jansen and colleagues treat advanced cervical cancer patients with Artenimol-R, hemi-succinate ester of DHA, for 28 days and find that Artenimol-R significantly alleviates cervical cancer and has no toxic effects. The mechanism of Artenimol-R improves cervical cancer is that Artenimol-R treatment results in significant reductions of p53, epidermal growth factor receptor (EGFR), Ki-67 (a marker of tumor proliferation) and angiogenesis (142). Deeken et al. recruit 19 patients with refractory solid tumors for a phase I clinical trial of ART and find that the maximum tolerated dose (MTD) of intravenous ART administration is 18mg/kg with a disease control rate of 27% (143). The above clinical studies provide the basis for the efficacy and safety of DHA in the treatment of malignant tumors.

In conclusion, DHA has demonstrated significant anti-tumor and inhibition of inflammation effects and is a promising drug with a wide range of targets. In recent years, studies on the genomics (64), proteomics (72, 113), metabolomics (58, 90) and network pharmacology (43) of DHA therapy have provided a basis for elucidating the specific pharmacological mechanism and targets of DHA, but there are few clinical studies on DHA. Further phase II and III clinical trials are required for DHA. To address the limitations and challenges of DHA in experimental research and clinical application, we consider that the mechanisms of DHA anti-tumor and inflammation inhibition need to be deeply elucidated, for example to explore the genetic or protein targets of DHA. Further, clinical trials of DHA as a therapeutic agent for tumor and inflammatory diseases need to be expanded. Although there have been few reports of serious adverse reactions to DHA, the safety of DHA in clinical use still needs to be monitored by clinicians. With the development of research on the pharmacological effects of DHA, it is certain that DHA will one day become an alternative treatment for tumors and inflammatory diseases.

## Author Contributions

RY: conceptualization, writing—original draft preparation and project administration. GJ: supervision, funding acquisition and writing—review and editing. MF: supervision and writing—review and editing. All authors contributed to the article and approved the submitted version.

## Funding

This study was sponsored by National Natural Science Foundation of China (No. 81960305, 61671098).

## Conflict of Interest

The authors declare that the research was conducted in the absence of any commercial or financial relationships that could be construed as a potential conflict of interest.

## Publisher’s Note

All claims expressed in this article are solely those of the authors and do not necessarily represent those of their affiliated organizations, or those of the publisher, the editors and the reviewers. Any product that may be evaluated in this article, or claim that may be made by its manufacturer, is not guaranteed or endorsed by the publisher.
